# Complete Genome Sequence of Buffalopox Virus

**DOI:** 10.1128/genomeA.00444-18

**Published:** 2018-05-24

**Authors:** Babak Afrough, Afia Zafar, Rumina Hasan, Roger Hewson

**Affiliations:** a National Infection Service Research, Public Health England, Porton Down, United Kingdom; b Department of Microbiology, Aga Khan University, Karachi, Pakistan

## Abstract

The first complete genome assembly of buffalopox virus isolate Karachi 2005, with a length of 195,630 bp, is presented here. Phylogenetic analysis shows the virus to cluster within Vaccinia species, and the genome contains 177 protein-coding sequences.

## GENOME ANNOUNCEMENT

Buffalopox virus (BPXV), family Poxviridae, genus Orthopoxvirus (OPV), is related to monkeypox, horsepox, variola, and cowpox viruses (1). BPXV causes mild to severe disease in buffaloes and cattle, presenting as pock lesions. BPXV is a highly transmissible zoonosis, and the disease in the Indian subcontinent has persisted for over 30 years following the cessation of human vaccination against smallpox virus. Clinical symptoms include high fever, unilateral axillary lymphadenopathy, and edema, often associated with the eruption of lesions which are normally confined to the hands, forehead, face, buttocks, and legs (2, 3). It is not normally transmitted between humans; however, during 5 months in 2004 to 2005, this virus caused a nosocomial outbreak in association with burn units in Karachi, Pakistan (4). BPXV continues to sporadically reemerge across central Asia and the Indian subcontinent (5–7), and a deeper understanding of the viral genome will contribute to improvements in surveillance and disease control.

BPXV isolate Karachi 2005 was passaged once in Vero cells (ECACC accession number 84113001), and the infectious titer of this passage was determined at 1.6 × 10^5^ PFU/ml by a viral plaque assay. Total DNA was isolated using the DNeasy kit (Qiagen), purified using Agencourt AMPure XP beads (Beckman Coulter), and quantified using a double-stranded DNA (dsDNA) broad-range (BR) assay on a Qubit 3.0 fluorometer (Life Technologies). Two hundred nanograms of DNA was used to prepare an SQK-LSK108-1D sequencing library (Oxford Nanopore Technologies) without shearing. MinION Nanopore sequencing (R9.4 chemistry) generated 248,398 base-called reads (Albacore version 2.1.10), with a median length of 1,844 bp. A draft assembly was obtained using Canu version 1.6 (8) as a single 193,093-bp supercontig. All other contigs and unassembled reads were screened against a locally curated OPV database to ensure that no orphan fragments were missed during the *de novo* assembly step. The draft genome was assessed using Quast version 4.6.3 (9) and refined using Nanopolish version 0.8.5 (10), where 1.56% of the Nanopore reads mapped to BPXV.

A Nextera XT paired-end library (Illumina) was prepared using 100 ng of the same BPXV culture supernatant and was sequenced on a HiSeq 2500 platform. This produced 6,378,742 reads, with 2.29% mapping to BPXV. The reads were processed with Trimmomatic version 0.36 (11), mapped to the draft 193,093-bp Nanopore genome using BWA (12, 13), and sorted using SAMtools version 1.7 (14). Correction and consensus calling with Illumina reads were performed with Pilon version 1.22 (15) on the draft genome, producing a final sequence of 195,630 bp. Annotation was performed with Glimmer version 3.02 (16), GeneMarkS version 4.28 (17), and protein BLAST searches.

Long-read Nanopore sequencing permitted the assembly of the genome as a single supercontig. This included intact characteristic OPV terminal inverted repeat (TIR) regions that flank the BPXV genomic termini. Nanopore sequencing alone made it difficult to identify and annotate many of the protein-coding genes with confidence; thus, Illumina data were used to supplement the Nanopore sequencing. The BPXV genome presents an average G+C content of 33.4% and contains at least 177 protein-coding genes. Whole-genome phylogenetic characterization using maximum likelihood approaches indicates chorioallantois vaccinia virus Ankara, the parental strain of modified vaccinia virus Ankara, to be its closest relative.

### Accession number(s).

The annotated genome sequence is deposited in GenBank under the accession number MG599038.
